# Advanced Atomic Layer Modulation Based Highly Homogeneous PtRu Precious Metals Alloy Thin Films

**DOI:** 10.1002/advs.202503561

**Published:** 2025-05-28

**Authors:** Yeseul Son, Sang Bok Kim, Debananda Mohapatra, Taehoon Cheon, Soo‐Hyun Kim

**Affiliations:** ^1^ Graduate School of Semiconductor Materials and Devices Engineering Ulsan National Institute of Science and Technology (UNIST) Ulju‐gun Ulsan 44919 Republic of Korea; ^2^ Center for Core Research Facilities Daegu Gyeongbuk Institute of Science and Technology (DGIST) Dalseong‐gun Daegu 42988 Republic of Korea; ^3^ Department of Materials Science and Engineering Ulsan National Institute of Science and Technology (UNIST) Ulju‐gun Ulsan 44919 Republic of Korea

**Keywords:** atomic layer deposition, atomic layer modulation, homogeneous composition, precious metals catalysis, PtRu atomic alloy

## Abstract

Atomic layer modulation (ALM) presents a novel approach for controlling the stoichiometry of platinum‐ruthenium (PtRu) alloys rather than a tedious atomic layer deposition (ALD) supercycling multielement ALD process. This method sequentially pulses dimethyl‐(*N,N*‐dimethyl‐3‐butene‐1‐amine‐*N*)platinum (C_8_H_19_NPt, DDAP) and tricarbonyl(trimethylenemethane)ruthenium [Ru(TMM)(CO)_3_] precursors with O_2_ as a counter reactant at 225 °C to produce ALM‐PtRu bimetallic alloys at the nanoscale. By smartly adjusting precursor pulsing times and temperatures, the average surface composition during growth can be modulated, achieving precise control over the PtRu alloy stoichiometry. Aberration‐corrected ultra‐high‐resolution scanning transmission electron microscope, Rutherford backscattered spectrometry, and advanced X‐ray diffraction analytical tools demonstrate homogenized Pt and Ru elemental distribution without localized segregation with adjustable Pt:Ru ratios ranging from 28:72 to 97:3. Demonstrating ≈100% step coverage on the high aspect ratio (≈30) 3D trench structures (top width of 125 nm, bottom width of 85 nm), the alloy maintains uniform thickness (≈30 nm) throughout its layers. ALM‐PtRu demonstrates durable and superior electrocatalytic performance compared to benchmark precious metal catalysts like ALD‐Pt and ALD‐Ru. This study highlights ALM's potential for precise alloy stoichiometry in PtRu films, offering significant promise for various applications, particularly electrocatalysis, and extending ALM to other metallic alloy systems.

## Graphical abstract

A unique idea of atomic layer modulation (ALM) that sequentially pulses DDAP and Ru(TMM)(CO)_3_, two precursors with one counter reactant O_2_ to produce ALM‐PtRu homogeneous PtRu bimetallic alloys at the nanoscale. It smartly adjusted precursor pulse time and temperature, ensuring stoichiometric and compositionally atomistic controlled highly polycrystalline conductive ALM‐PtRu thin films achieved 100% step coverage on the high aspect ratio 3D trench structures.

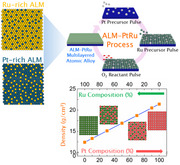

## 1 Introduction

Thin film alloys have enhanced physicochemical electrical properties compared to their single metallic component, eliminating the limitation of single atoms/elements. Specifically, they have higher strength and durability than single metals and are more corrosion and abrasion‐resistant in high‐temperature environments.^[^ ^1^ ^]^ These alloys can increase reaction kinetics and selectivity when used as catalysts, facilitating efficient reactions in the electrochemical water‐splitting process for a sustainable hydrogen economy.^[^ ^2^ ^]^ Additionally, given the current downsizing of electronic devices, especially for the CMOS technology, impurity‐free, homogeneous, atomic‐scale binary precious metal alloys are required for gate electrode applications to control the electrode work function.^[^ ^3^ ^]^ These bi‐metallic thin films would be essential components in advanced electronic architectures,^[^ ^4^ ^]^ where precise control over film thickness and uniform coverage on complex, high‐aspect‐ratio structures is critical for ensuring reliable electrical characteristics.^[^ ^5^ ^]^ ALD is an industrially preferred method for fabricating these thin films, enabling precise control of atomic thickness and composition, credited to their self‐limiting surface reactions by sequentially exposing precursors and reactants to a surface.^[^ ^6^ ^]^

ALD has the potential to make alloys using the supercycle concept, where two or more ALD processes are used sequentially and repeatedly to form compound or alloy films. By adjusting the ratio of individual ALD cycles, called subcycles, the composition of the deposited alloy thin film can be controlled, which in turn allows for the regulation of the resulting alloy thin film's properties and performance.^[^ ^7^ ^]^ Particularly in the case of forming metal alloys, two or more metal ALD processes are performed alternately, allowing for the adjustment of the ratio of specific metals or the creation of the desired alloy composition and homogeneity.^[^ ^8^ ^]^ However, since each metal is deposited through independent ALD sub‐cycles, there is a possibility that the metals are initially stacked nano‐laminate structure in a spatially separated manner, which may hinder the formation of a homogeneous atomic alloy structure on later stage^[^ ^9^ ^]^ As a result, post‐deposition annealing or elevated processing temperatures are often required to promote interdiffusion and alloying. In addition, the need to repeat separate ALD cycles for each precursor leads to prolonged process times and increased precursor consumption, thereby reducing the overall efficiency of the supercycle approach. Therefore, the ALM method is proposed to form a single‐layer multicomponent thin film by sequentially pulsing two precursors and then reacting with only one reactant to overcome these limitations. The core concept of ALM is that the composition ratio is determined by the two precursors' steric hindrance and chemical reactivity based on the ligand size and adsorption sites. This ALM method mixes two or more types of elements on the target surface so it can deposit a uniform, homogeneous alloy at the atomic scale. A technique to form thin films of oxide alloy using ALM was recently proposed. For RuAlO thin films, the compositional differences were observed according to the pulsing order of two precursors with different ligand/precursor sizes. Changing the pulsing order of the precursors significantly altered the (Al/(Al+Ru)) ratio from 31% to 89%.^[^ ^10^ ^]^ Similarly, in the case of ZrHfO_x_ thin films, varying the precursor pulsing sequence resulted in distinct compositions, with the Zr/(Zr+Hf) ratio changing from 66% to 54%.^[^ ^11^ ^]^ These compositional variations were explained by differences in surface chemical adsorption and steric effects, highlighting the critical role of precursor pulsing order in determining film composition. Another approach for controlling composition is to adjust the relative amount of adsorbed precursors, especially when the precursors are similar in size. Key parameters in this process include precursor pulsing time or exposure time. Increasing the precursor pulse time allows more precursor molecules to be injected, which increases the amount adsorbed on the surface.^[^ ^12^ ^]^ In addition, the exposure time is related to the time that the precursor remains on the surface and is controlled by factors such as the pressure inside the chamber and the precursor concentration.^[^ ^13^ ^]^ The Langmuir adsorption model can be used to predict the surface saturation state, and based on this, the exposure time can be set so that a specific element is fully or partially adsorbed on the surface. This way, various composition ratios can be implemented by controlling the surface saturation and unsaturation state. Another method for composition control involves adjusting the precursor vapor pressure. Notably, the vapor pressure is a significant factor determining how easily the precursor vaporizes, affecting the rate at which the precursor reaches the surface and the amount of adsorption.^[^ ^14^ ^]^ The precursors with high vapor pressure readily volatilize, rapidly diffusing within the chamber and reaching the surface. This allows the precise control of the composition and homogeneity of compounds and alloys to be controlled in the ALM process.

This study strategically adopts a PtRu model system for the ALM process, as Pt and Ru processes have been actively addressed in ALD research due to their unique physicochemical, electrical, and electrochemical properties. For instance, Pt has been widely used in fuel cells, electrochemical sensors, hydrogen production systems, etc., due to its excellent catalytic properties and durability, and much research intended to improve the catalytic activity and uniformity of thin films.^[^ ^15^ ^]^ On the other hand, Ru has been studied for its high electrical conductivity, oxidation resistance, and benchmark catalytic activity even at low temperatures.^[^ ^16^ ^]^ By combining the advantages of these individual precious metals, ALM‐PtRu alloys can simultaneously exhibit the synergetic properties of both materials. In the current ALM process, Pt and Ru precursors are alternately used, reacting with a single oxygen reactant to deposit ALM‐PtRu films, as most Pt and Ru ALD processes use oxygen as the reactant in a typical ALD window of 200∼300 °C.^[^ ^6^, ^17^ ^]^ Plus, as noble metals, Pt and Ru are mainly unaffected by the physical/chemical state of the substrate, even in high‐temperature environments, enabling consistent thin film deposition on various substrates.^[^ ^18^ ^]^ Due to these intrinsic characteristics, ALM facilitates meticulous regulation of the atomic composition of PtRu films, establishing it as an appropriate methodology for producing homogeneous, high‐quality thin films. In this investigation, PtRu films were synthesized utilizing ALM, wherein the alloy composition was manipulated by varying the quantity of precursor adsorbed onto the substrate. Overall, the precursor amount was regulated through two approaches: varying the precursor pulsing time or modifying the precursor source temperature. This is schematically illustrated in Figure S1 (Supporting Information), which provides a simplified depiction of the process. The formation of alloy films begins with the exposure of Pt precursor under conditions where its adsorption is deliberately limited—either by using a short pulsing time or by lowering the source temperature below its optimal value. This results in a reduced chemisorption of Pt precursor compared to Ru precursor, promoting the formation of a Ru‐rich alloy. In the subsequent step, Ru precursor is introduced to the surface, achieving saturation via its self‐limiting chemisorption mechanism. The O_2_ reactant is then introduced to remove ligands from the chemisorbed species of both precursors, enabling atomic mixing of Pt and Ru at the surface and forming a homogeneous alloy film enriched in element Ru. Conversely, a Pt‐rich alloy can be achieved by enhancing the relative adsorption of the Pt precursor. This is accomplished by increasing the pulsing time or optimizing the source temperature of the Pt precursor to achieve a higher chemisorption than the Ru precursor. Following this step, the Ru precursor is introduced and allowed to reach surface saturation. Ligand removal and subsequent atomic mixing occur through reaction with the reactant, resulting in a thin film enriched in Pt element. This process demonstrates the versatility of the ALM approach, which leverages the sequential and self‐limiting nature of ALD chemisorption to control the composition and uniformity of alloy films precisely. The resulting ALM‐PtRu films were analyzed through density measurements obtained by X‐ray Reflectivity (XRR), which exhibited a strong correlation with the composition determined via Rutherford Backscattering Spectrometry (RBS). Furthermore, consistent deposition of Pt and Ru was validated across all areas (top, middle, bottom) of a 3D trench wafer (aspect ratio ∼30). Beyond the PtRu system, the current ALM technique can be broadened to encompass the deposition of compound and alloy films at the atomic scale, indicating significant potential for electrocatalysis and electronic device applications.

## 2 Results And Discussion

### 2.1 Growth Kinetics of ALD‐Pt and ALD‐Ru Processes

**Figure 1:**
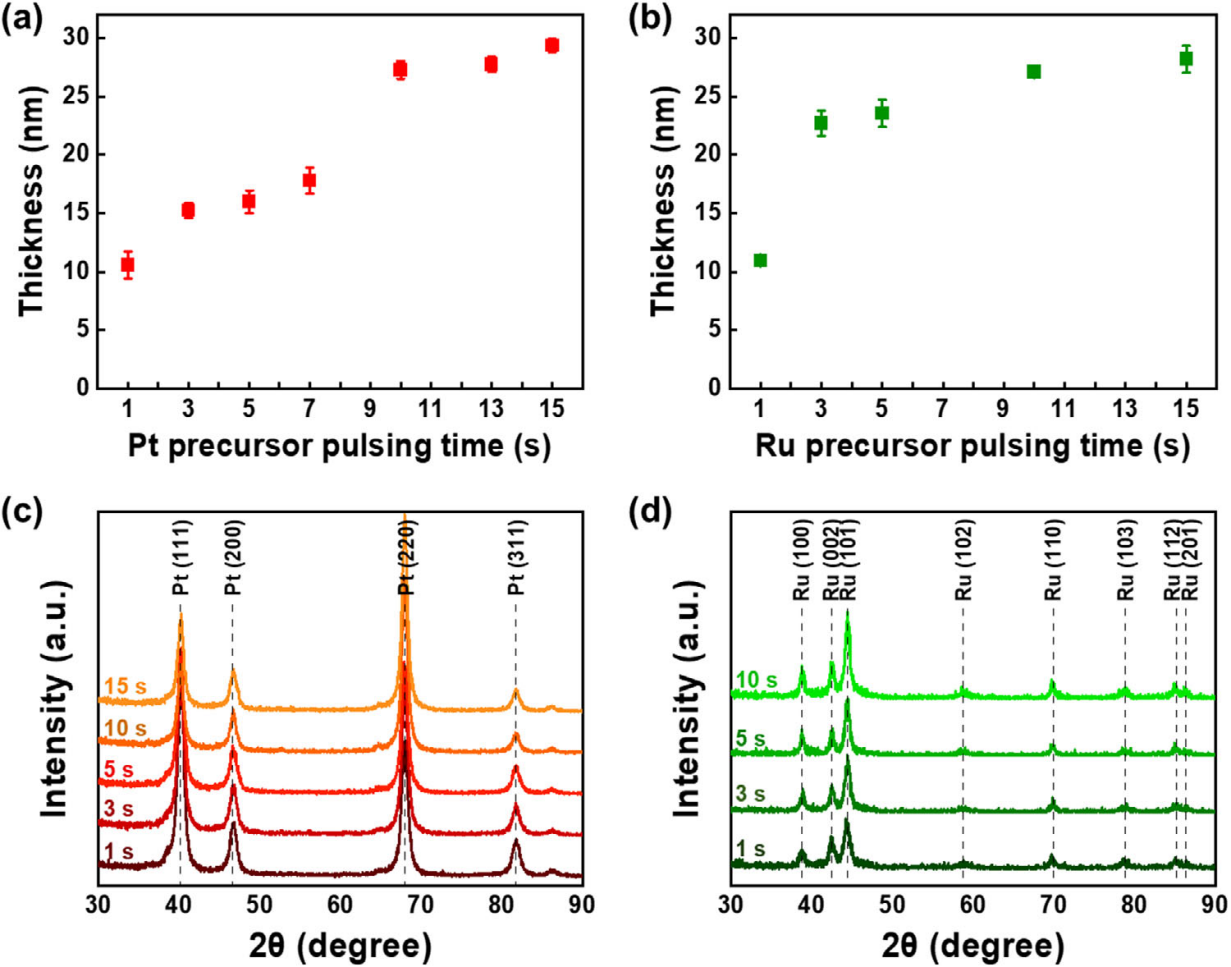
Saturated and non‐saturated growth processes of ALD‐Pt and ALD‐Ru, and associated self‐saturation growth factors. a) Thickness and c) XRD patterns of ALD‐Pt films as a function of Pt precursor pulsing time ranging from 1 to 15 s. b) Thickness and d) XRD patterns of ALD‐Ru films as a function of Ru precursor pulsing time ranging from 1 to 15 s.

Before the ALM‐PtRu process, the self‐saturated growth behaviors of ALD‐Pt and ALD‐Ru were evaluated at 225 °C using a fixed number of 200 ALD cycles. **Figure** **1**a,b show the self‐saturated growth of the film growth as a function of Pt and Ru pulsing time, respectively. In the case of Pt precursor, the average film thickness kept increasing to a pulsing time from 1 to 15 s. However, when the pulsing time exceeded 10 s, surface saturation due to a self‐limiting reaction occurred, and no significant further increase in film thickness was observed (Figure 1a). For the Ru precursor, the average thickness of the thin films increased with increasing precursor pulse time from 1 to 3 s, but it exhibits self‐saturated growth behavior above 3 s (Figure 1b). It is crucial to emphasize that when the precursor pulsing time is short and the process is performed under nonsaturated conditions, the film thickness is thinner than that grown under saturated conditions. This is because the amount of precursor adsorbed on the surface is not sufficiently saturated, and this phenomenon can vary depending on the vapor pressure of the precursor, equipment conditions, temperature, and processing pressure. Such unsaturated growth is a typical feature observed across all ALD processes, including metals, oxides, sulfides, and nitrides.^[^ ^17^, ^19^ ^]^ In this context, the difference in self‐saturated growth between ALD‐Pt and ALD‐Ru can be attributed to the combined effects of vapor pressure and precursor size. The Ru precursor, with a higher vapor pressure and slightly smaller molecular size than the Pt precursor, allows for more rapid and efficient delivery of precursor molecules to the substrate surface. This allows more Ru precursor to be adsorbed on the surface in a shorter time, facilitating typical ALD self‐limited growth behavior and, thus, faster surface saturation. On the other hand, the Pt precursor with lower vapor pressure delivers fewer molecules to the surface, resulting in a slower saturation process. Grazing‐incidence angle X‐ray diffraction (GIAXRD) analysis was performed (Figure 1c,d) to investigate the crystallinity of ALD‐Pt and ALD‐Ru films as a function of precursor pulsing time. Figure 1c presents the GIAXRD patterns of ALD‐Pt films deposited with varying Pt precursor pulsing times. The XRD analysis was conducted within a 2θ range of 30°–90° for Pt films grown on SiO₂/Si substrates. The diffraction peaks observed at 2θ = 39.76°, 46.24°, 67.45°, 81.29°, and 85.71° correspond to the (111), (200), (220), and (311) planes of face‐centered cubic (FCC) Pt, consistent with the reference JCPDS data (Ref. No. 00‐004‐0802). The crystallinity improved with increasing pulsing time of the Pt precursor, especially in the (111) and (200) directions, which are characteristic of the dense and close‐packed planes in the FCC structure of Pt. Figure 1d shows the GIAXRD results of ALD‐Ru films deposited with varying Ru(TMM)(CO)_3_ pulsing times, also within the 2θ range of 30°–90°. The diffraction peaks at 2θ = 38.53°, 42.30°, 44.17°, 58.54°, 69.70°, 78.68°, 85.04°, and 86.32° correspond to the (100), (002), (101), (102), (110), (103), (112), and (201) planes of hexagonal close‐packed (HCP) Ru, by the JCPDS reference (Ref. No. 01‐073‐7011). These peaks confirmed the formation of the HCP‐Ru crystal phase and showed similar crystallinity during all the Ru precursor pulse times. In this study, Pt, which has an extended unsaturated region compared to Ru, was introduced as the first precursor in the ALM process, and the properties of the ALM‐PtRu films were analyzed by adjusting the pulse time of the Pt precursor to 1, 3, 5, and 10 s (sections 2.2 and 2.3).

### 2.2 ALM‐PtRu Process via the Control of the Precursor Pulsing Time

**Figure 2:**
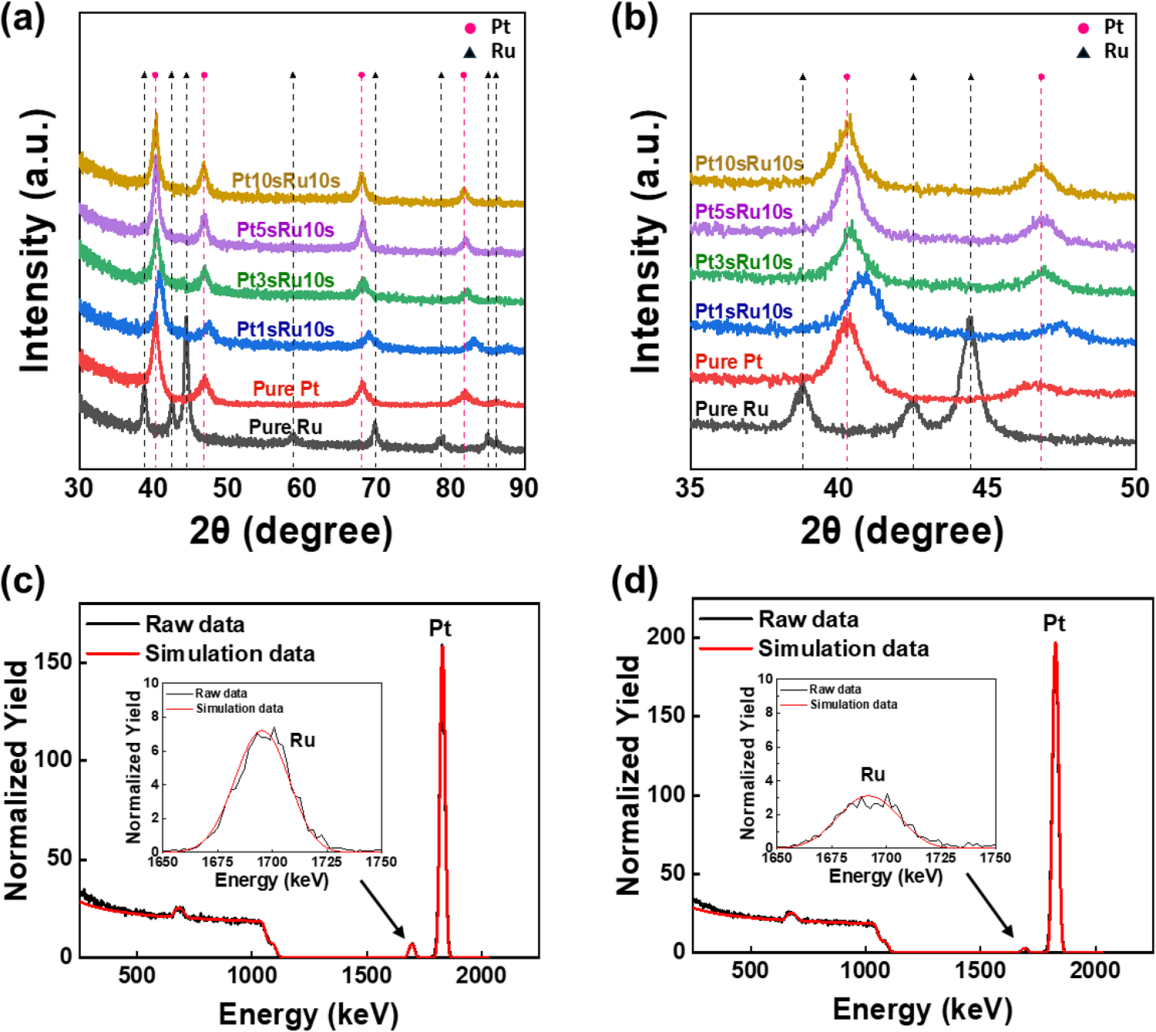
XRD patterns of ALM‐PtRu films as a function of Pt pulsing time: a) over the 2θ range of 30°–90°, and b) in the 2θ range from 35°–50° highlighting the shift of the Ru peak toward the Pt peak with increasing Pt content. RBS analysis results of ALM‐PtRu films as a function of Pt precursor pulsing time: c) 3‐seconds and d) 10‐seconds pulsing.

**Figure** **2**a,b show the XRD results of the films grown by the ALM process as a function of the pulse time of the Pt precursor, which was provided first during the ALM process. The samples for XRD analysis include PtRu films deposited with Pt precursor pulsing times of 1, 3, 5, and 10 s, as well as Pt and Ru films grown by the ALD process of each metal separately for comparison. For comparison, the XRD results of Ru and Pt single metal films grown by each ALD process are also shown. XRD analysis allows us to characterize the phase and crystallinity of the deposited films and understand the effect of the relative amount of two precursors adsorbed on the surface on the phase and crystallinity of the deposited film. The Pt‐Ru binary alloy system exhibits an FCC solid solution phase up to ≈60 at.% Ru, and a HCP solid solution phase above ≈80 at.% Ru.^[^ ^20^ ^]^ As shown in Figure 2a, the XRD analysis of the pure Pt film showed a typical FCC structure (space group: Fm‐3m, 225) characterized (111), (200), (220), and (311) planes, whereas the pure Ru film showed an HCP structure (space group: P63/mmc, 194) with (100), (002), (101), (102), (110), (103), (112), and (201) planes. Despite the Pt precursor pulsing time, deposited films generally exhibited peaks corresponding to the Pt FCC structure, indicating that the films consist of Pt‐Ru alloy at the nanoscale. Although Pt and Ru have different crystal structures (Pt has an FCC structure and Ru has an HCP structure), the atomic arrangement of the Ru (002) plane is identical to that of the Pt (111) plane. Therefore, the interplanar spacing of these planes was used as an indicator of the formation process of the alloy film, which is shown in Figure 2b. In the pure Ru film, the (002) peak appeared at 42.2°, whereas in the pure Pt film, the (111) peak appeared at 39.8°. As the Pt precursor pulsing time increased from 1 to 10 s during the ALM process, one can consider that the relative amount of Pt precursor absorbed would continuously increase, and the space necessary for the adsorption of the Ru precursor decreased. Correspondingly, XRD peaks were shifted to lower 2θ angles, moving closer to the Pt (111) peak. Notably, the film prepared with a 10‐second Pt precursor pulsing time exhibited a peak identical to that of pure Pt. This peak shift indicates that the Ru and Pt atoms in the deposited films were mutually substituted in the crystal lattice, forming a solid solution with a changed lattice constant. This peak shift reflects the lattice distortion caused by the interaction between Pt and Ru, suggesting that the two metals were uniformly mixed to form a homogeneous atomic alloy.^[^ ^3^, ^21^ ^]^

RBS analysis was performed to determine the precise composition of the PtRu films prepared by the ALM process. Figure 2c,d show the RBS spectra of the Pt 3‐, 10‐second pulsed PtRu films. Figure 2c shows the result of the Pt 3‐second pulsed PtRu film, where the backscattering signals of both Pt (≈1830 keV) and Ru (≈1700 keV) were observed when the incident He^++^ energy is 2 MeV. The composition ratio of Pt to Ru was 87:13 based on the analysis results. The density of the ALM‐PtRu films was also calculated using the area density, film thickness, and composition and was found to be 19.64 g cm^−^^3^. This value is between the bulk Pt density (21.4 g cm^−^^3^) and the bulk Ru density (12.4 g cm^−^^3^), suggesting Ru incorporation into FCC Pt with the formation of Pt‐Ru homogeneous alloy at the nanoscale. Figure 2d shows the result of the Pt 10‐second pulsed PtRu film. The backscattering signals of both Pt (≈1830 keV) and Ru (≈1700 keV) were observed. The backscattering signal of Ru shows a lower normalized yield compared to Figure 2c. The composition ratio of Pt to Ru was 95:5, according to the analysis results. The density of the ALM‐PtRu film calculated based on the RBS data is 20.65 g cm^−^^3^, which is similar to the bulk Pt density value. Impurity levels were analyzed using time‐of‐flight elastic recoil detection (ToF‐ERD) analysis to evaluate the purity of the alloy films. Figure S2 (Supporting Information) shows the results of further characterizing the impurities such as C and O remaining in the Pt 3‐second pulsed PtRu film and Pt 10‐second pulsed PtRu film by ToF‐ERD analysis. The analysis results show that the C and O contents of the Pt 3 s‐pulsed PtRu alloy film are 0.01 at.% and 0.15 at.%, respectively, and those of the Pt 10‐s pulsed PtRu alloy film are 0.01 at.% and 0.18 at.%, respectively. Considering that the C and O contents of the ALD‐Ru film using [Ru(TMM)(CO_3_)] precursor are 0.01 at.% and 0.03 at.%,^[^ ^6b^ ^]^ respectively, the impurity contents of the PtRu alloy film are still maintained at a low level. In addition, negligible impurities were detected in the ALD‐Pt film using DDAP precursor.^[^ ^17b^ ^]^ This suggests that the characteristics of impurities observed in the ALD‐Pt and ALD‐Ru processes were also reflected in the PtRu alloy films prepared by the ALM process, suggesting that the excellent reactivity of both Pt and Ru precursors with O_2_ as a reactant contributed to maintaining a low impurity content in the alloy.

**Figure 3:**
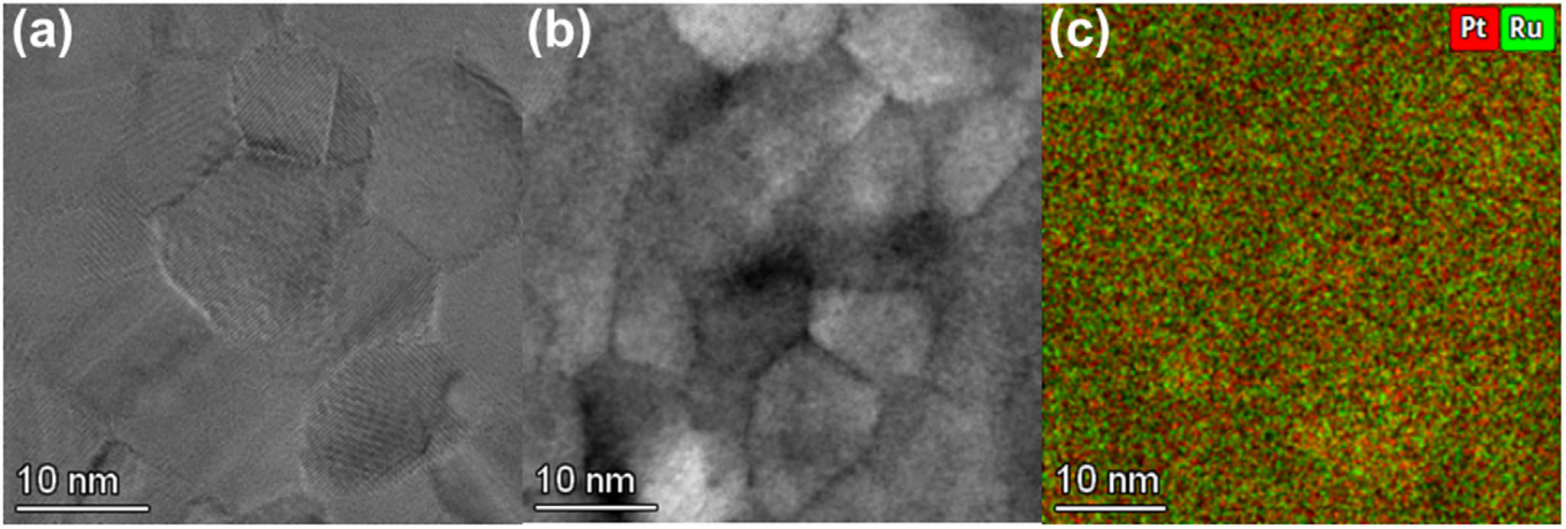
Plan view of ALM‐PtRu films showing a) HRTEM images, b) STEM‐BF images, and c) EDS mapping images of the ALM‐PtRu film deposited on a SiO_2_/Si substrate.

We performed plan‐view TEM analysis to evaluate the microstructure and compositional uniformity of the fabricated Pt 1‐second pulsed PtRu film (**Figure** **3**). In Figure 3a, the plan‐view high‐resolution TEM (HRTEM) image shows a polycrystalline structure with well‐defined lattice fringes, showing randomly oriented atomic arrangements. The selected area diffraction pattern (SADP) in Figure S3a (Supporting Information) exhibits discontinuous spots arranged in concentric rings, a characteristic of polycrystalline materials. These diffraction spots correspond to FCC structure planes such as (111), (200), (220), and (311), with spacings that reflect the lattice distortion caused by Pt and Ru alloying. This finding demonstrates that the FCC structure predominantly persists locally, while the XRD analysis reflects the average structure, indicating peaks between FCC and HCP phases. The SADP results confirm that the FCC symmetry extends over a broader area, suggesting that it is not confined to localized regions. The scanning transmission electron microscopy bright‐field (STEM‐BF) image reveals a clear contrast among the grains due to variations in orientation and thickness, highlighting the polycrystalline nature of the alloy (Figure 3b). This diffraction contrast emphasizes the presence of randomly oriented nanocrystalline grains, consistent with the random orientations observed in the SADP. Additionally, BF‐TEM imaging (Figure S3b, Supporting Information) reveals an average grain size to be ≈11 nm. The dark‐field TEM (DF‐TEM) image (Figure S3c, Supporting Information) selectively emphasizes grains satisfying specific Bragg conditions, confirming the uniform crystallinity and size distribution, as well as the random orientations and polycrystalline arrangement of the grains. The high diffraction contrast in the DF‐TEM image further validates the crystallinity observed in the HRTEM analysis. The compositional uniformity of the ALM‐PtRu alloy was evaluated via STEM energy‐dispersive X‐ray spectroscopy (EDS) mapping (Figure 3c), which clearly shows the homogeneous distribution of Pt (red) and Ru (green) within individual grains. The EDS mapping results reveal a Pt‐to‐Ru ratio of ≈62:38, highlighting the uniform mixing of Pt and Ru at the nanoscale. Moreover, the EDS‐derived composition aligns with the density‐based composition calculated from XRR, reinforcing the reliability of compositional control in the ALM process. This consistency indicates that the Pt and Ru elements maintain uniform mixing in terms of both composition and density, confirming the formation of a structurally and chemically homogeneous alloy. Together, these results validate the successful synthesis of a uniformly alloyed PtRu thin film through the ALM process.

**Figure 4:**
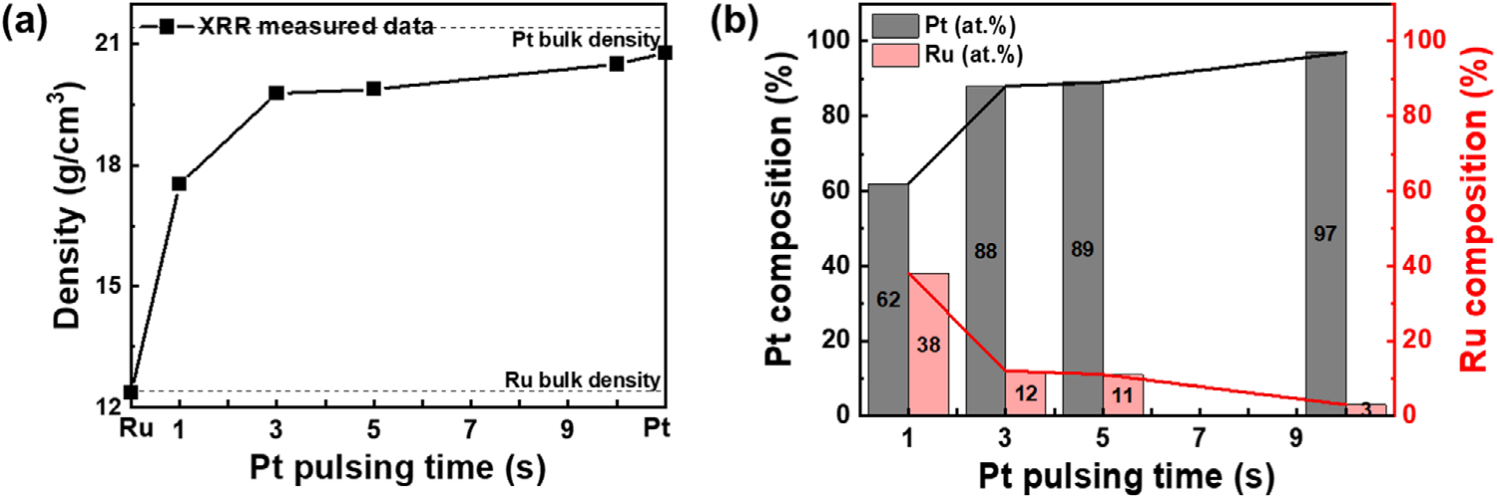
a) The density of ALM‐PtRu films on SiO_2_/Si substrate as a function of Pt precursor pulsing time from XRR analysis, with b) the corresponding Pt and Ru composition calculated from the density measured by XRR.

From the RBS analysis, the correlation between the composition of alloy films and their densities is apparent because there is a big difference between the bulk density of Pt and that of Ru. So, the density of alloy films can be an indicator for the composition and the compositions of the alloy films can be calculated from their densities. **Figure** **4** shows the density of the ALM‐PtRu films according to the Pt precursor pulsing time and the alloy composition results calculated using it. The density of the films was obtained from XRR analysis at angles above which X‐rays start to penetrate the film. The XRR raw data and their simulation are shown in Figure S4 (Supporting Information). In Figure 4a, the density of the Pt 1‐second pulsed PtRu film was determined to be 17.54 g cm^−3^, which is between the measured bulk Pt density (20.77 g cm^−3^) and bulk Ru density (12.36 g cm^−3^). The densities of the Pt 3‐, 5‐second pulsed PtRu films were determined to be 19.77 and 19.87 g cm^−3^, respectively, from the simulation of XRR data. These values are higher than that Pt 1‐second pulsed PtRu film because the density increased due to the increase in Pt content with a higher atomic mass than that of Ru as the Pt pulsing time increased. The density of Pt 10‐second pulsed PtRu film was measured to be 20.5 g cm^−3^, which is very similar to the bulk Pt value. This suggests that the initially pulsed Pt was saturated, forming an alloy with a minimal Ru content. In Figure 4b, the composition of the PtRu films was calculated using the density measured by XRR analysis. The compositions of the PtRu films, with Pt pulsing times of 1, 3, 5, and 10 s, were determined to be 62:38, 88:12, 89:11, and 97:3, respectively. The composition calculated from the XRR‐measured density for the Pt 1‐second pulsed PtRu film is 62:38, identical to that obtained from STEM‐EDS analysis (Figure 3c). For the Pt 3‐second and 10‐second pulsed films, the compositions determined by XRR are 88:12 and 97:3, respectively, showing only minor discrepancies of ≈2 at.% compared to the RBS results (87:13 and 95:5). These close agreements across the techniques highlight that the composition of ALM‐PtRu films can be reliably and accurately inferred from the XRR‐measured density. An illustration of this is shown in Figure S5 (Supporting Information). The Y‐axis represents density, and the X‐axis represents the composition of Pt and Ru, and the relationship between the two variables is expressed in the form of a linear function. Suppose the density measured by XRR is similar to this linear function. In that case, it means that the composition is close to the trend line of the linear function, indicating consistency between the compositions. This consistency suggests that the microstructure and composition of the alloy are within the predictable range, emphasizing that the density obtained by the XRR analysis is a reliable indicator of the composition.

### 2.3 ALM‐PtRu Process via the Control of the Precursor Temperature

Method 1, as described in the previous section, effectively controlled the PtRu alloy composition, though the resulting films exhibited a Pt‐rich composition. Meanwhile, method two was introduced, aiming to reduce the Pt content by lowering the temperature of the Pt precursor to limit its adsorption. To expand the range of composition control, Figure S6 (Supporting Information) shows the growth per cycle (GPC) achieved at two Pt precursor temperatures using the same pulsing time. The pulsing times of the Pt precursor were set to 1, 3, 5, and 10 s, and a decreasing trend of GPC with decreasing precursor temperature was observed at all pulsing times. These results indicate that lowering the temperature of the Pt precursor decreases the vapor pressure, reducing the amount of Pt precursor adsorbed onto the substrate and thus decreasing the GPC. Similar trends have been observed in other ALD processes, where lowering the precursor temperature reduces the vapor pressure, decreasing the amount of precursor adsorbed onto the substrate.^[^ ^22^ ^]^ This, in turn, results in a reduced GPC, as fewer precursors are available for deposition during each cycle. Based on these results, we optimized the alloy composition by adjusting the processing conditions to decrease Pt adsorption in the PtRu alloy films. This approach illustrates that precursor temperature control not only influences the GPC but also enhances composition adjustment. By minimizing Pt adsorption, we achieved greater flexibility in fine‐tuning the stoichiometry of the PtRu films. These findings underscore the significance of adjusting processing conditions to enable precise composition control in alloy fabrication.

**Figure 5:**
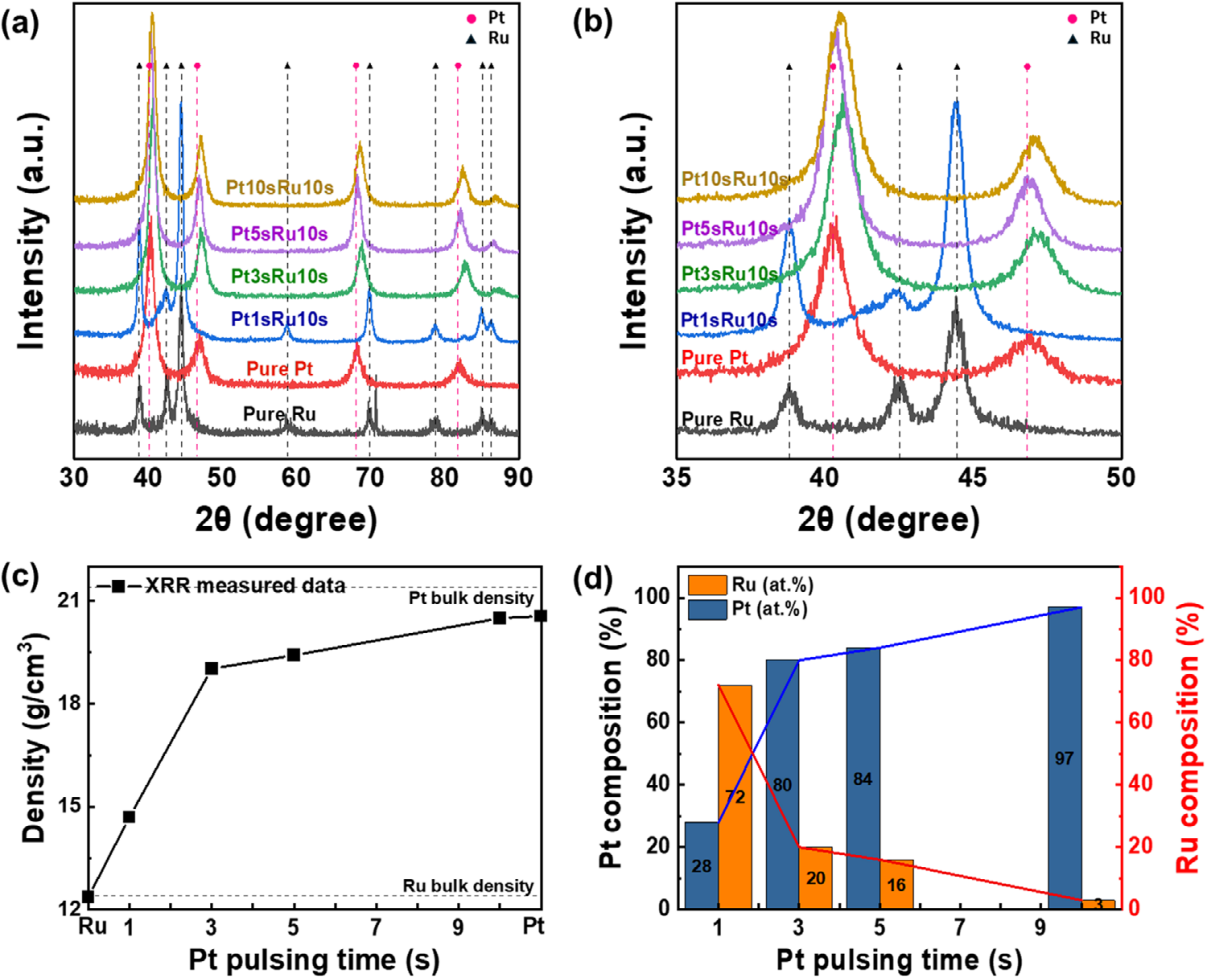
XRD patterns of ALM‐PtRu films as a function of Pt pulsing time, shown a) over the 2θ range of 30°–90° and b) in the 2θ range of 35°–50°. c) The density of ALM‐PtRu films obtained from XRR analysis as a function of Pt precursor pulsing time, with d) the corresponding Pt and Ru composition calculated from the density.

**Figure** **5**a,b show the XRD analysis of the PtRu film formed using method two. Figure 5a shows the XRD pattern for the 2θ range from 30° to 90°, with the Pt 1‐second pulsed PtRu film displaying a prominent peak associated with the HCP structure of Ru. In this sample, the XRD pattern shows overlapping peaks near 2θ ≈ 42°, where the Ru (002) reflection from its HCP structure coincides with the FCC structure's Pt (111) reflection. This overlap, coupled with the film's low crystallinity, results in broad, low‐intensity peaks suggestive of structural disorder or the presence of overlapping phases rather than distinct crystalline domains. Additionally, a faint signal 2θ ≈ 82°, attributed to the Pt (311) plane, is observed but subdued, likely due to lower Pt content and the dominant influence of the Ru matrix on the crystal structure. The Ru composition of this sample is 72 at.% (Figure 5d), which is slightly below the ∼80 at.% threshold typically required for stable HCP phase formation according to phase diagrams. Nevertheless, the short Pt pulse time and low deposition temperature may have hindered Pt crystallization, favoring its substitutional incorporation into the Ru lattice. This indicates that Ru can maintain its HCP structure at certain compositions and temperatures even when alloyed with small amounts of Pt. The initial dominance of the Ru peak suggests minimal Pt incorporation into the crystal structure, confirming Ru as the primary phase. As the Pt precursor pulse time increases, the peak in Figure 5a gradually shifts toward positions associated with Pt, indicating greater Pt atom incorporation.

To highlight this shift in detail, Figure 5b provides an enlarged view of the XRD pattern within the 2θ range of 35° to 50°. This closer examination reveals that, with increased Pt pulsing, the peak position progressively moves to lower 2θ angles, suggesting a lattice expansion due to the introduction of larger Pt atoms into the Ru lattice. XRR analyzed the alloy's density to infer the PtRu alloy's composition (Figure 5c). The density of the Pt 1‐second pulsed PtRu film was 14.7 g cm^−3^, which was a bit higher than that of the bulk Ru density (12.4 g cm^−3^). This can be attributed to the limited adsorption of Pt on the substrate, likely due to its relatively low vapor pressure, which results in a lower deposition rate for Pt compared to Ru. Consequently, the film's composition becomes dominated by Ru, leading to a density closer to that of bulk Ru. The densities of the Pt 3‐, 5‐second pulsed PtRu films were similar (19.03 and 19.42 g cm^−3^), though it is slightly increased with increasing Pt precursor pulsing time. For the Pt 10‐second pulsed PtRu film, the density approached that of bulk Pt, indicating that the Pt precursor reached saturation during the deposition process (Figure S7, Supporting Information). This suggests that the amount of Ru incorporated into the film was relatively low, resulting in the formation of a Pt‐rich alloy. The similarity in density to bulk Pt is consistent with a higher concentration of Pt in the film, which is indicative of a phase with minimal Ru substitution, possibly leading to a Pt‐rich solid solution or phase separation with a low Ru content. Figure 5d presents the composition of PtRu films, calculated from XRR density, as a function of Pt precursor pulsing time. When the Pt precursor temperature was set to 55 °C, varying the pulsing time to 1, 3, 5, and 10 s resulted in Pt:Ru ratios of 28:72, 80:20, 84:16, and 97:3, respectively. This trend is due to the increased adsorption of Pt precursor as the pulsing time increases, leading to a higher Pt content in the alloy. In method one, setting the Pt precursor temperature to 65 °C enhances the precursor vapor pressure, enabling efficient Pt adsorption even at shorter pulsing times. This condition accelerates the increase in Pt content, making it suitable for applications requiring Pt‐rich alloy compositions. In contrast, method two utilizes a lower precursor temperature, allowing for control over the alloy composition across a broader range and accommodating various compositional requirements. These findings confirm that adjusting the precursor pulsing time and temperature enables precise alloy film composition control using the ALM technique. This demonstrates its adaptability to tailor compositional ratios to meet diverse application needs.

**Figure 6:**
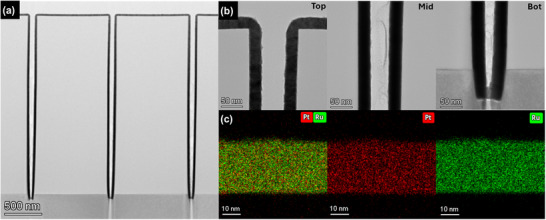
a) BF‐TEM image of ALM‐PtRu film showing overall step coverage, b) step coverage analysis at the top, middle, and bottom sections, and c) EDS mapping of Pt and Ru in the top section of the film on a 3D trench wafer.

We employed a Pt 1‐second pulsed PtRu film deposited via ALM on a patterned wafer with a 3D structure (aspect ratio of ≈30, top width of 125 nm, and bottom width of 85 nm) to examine the conformality of PtRu films. **Figure** **6**a shows the step coverage analysis of the PtRu alloy deposited on the patterned wafer. The measured thicknesses at the top, middle, and bottom were measured as 28.1, 29.7, and 29.3 nm, respectively. The sidewall and bottom step coverage reached ≈100%, and the similarity in thickness suggests that the PtRu alloy grows uniformly across the entire 3D structure. The high‐angle annular dark‐field STEM (HAADF‐STEM) image in Figure S8a (Supporting Information) further demonstrates the physical conformality of the PtRu film, as shown by the consistent thickness along the trench sidewalls and bottom. Additionally, the EDS mapping results for Pt (Figure S8b, Supporting Information) and Ru (Figure S8c, Supporting Information) confirm the effective penetration of the alloy film, achieving uniform coverage down to the bottom of the high aspect ratio structure. As shown in Figure 6b, the BF‐TEM images of the trench at the top, middle, and bottom reveal continuous film formation in each region. The surface is smooth and maintains a consistent thickness, confirming the uniform deposition of the PtRu film throughout the trench. The EDS mapping in Figure 6c shows the distribution of Pt (red) and Ru (green) at the top of the 3D trench wafer, confirming their consistent presence and homogeneous mixing at the nanoscale. In contrast to the commonly used supercycle method for manufacturing and fabricating nano‐laminate structures (Figure S9, Supporting Information), which consists of alternating ALD‐Pt and ALD‐Ru subcycles in a 3:7 ratio, the ALM process results in PtRu films that maintain high homogeneity. These results indicate that the ALM process effectively preserves both thickness and compositional uniformity, enabling the formation of stable PtRu alloy films. The method ensures both physical and compositional uniformity in high‐aspect‐ratio structures. This excellent step coverage and uniformity, achieved through precise deposition, can be attributed to the saturation reaction and sequential deposition mechanism inherent in ALD processes. These advantages are effectively retained in the ALM process, allowing for uniform alloy mixing and consistent film formation throughout the structure, even in high‐aspect‐ratio structures.

**Figure 7:**
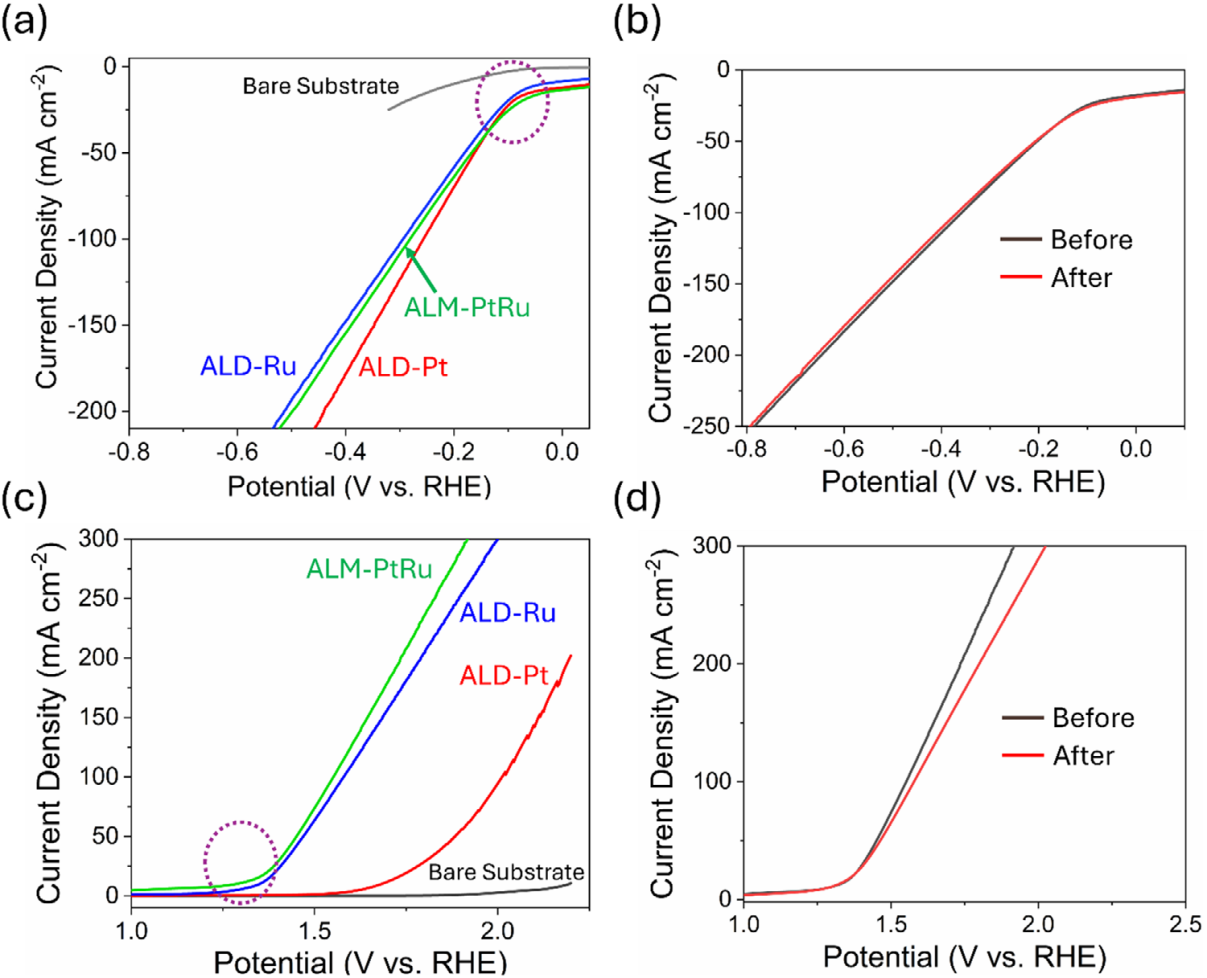
Electrocatalysis activities and stability for designed ALM‐PtRu thin film alloy, ALD‐Pt, and ALD‐Ru thin films. a) HER, c) OER LSV curves and long‐term durability screening for ALM‐PtRu thin film alloy for b) HER and d) OER catalytic processes.

Considering the distinct crystallinity, electrical and chemical properties of bimetallic homogeneous ALM‐PtRu thin films and those of their parent elemental ALD‐Pt and ALD‐Ru processes, we investigated all three catalysts in **Figure** **7** for hydrogen/oxygen evolution reaction (HER/OER) electrochemical activities and durability. The idea is to explore and introduce an early prototype of electrocatalysts fabricated from homogeneous ALM‐PtRu atomic alloys by extending their versatility to other precious metal atomic alloys. As precious metals are rare and expensive, their precise use is paramount compared to their bulk counterparts, primarily used as benchmark catalysts in various catalysis applications. An atomic‐scale bimetallic alloy such as ALM‐PtRu could simultaneously slow the consumption of precious metals in the impurity‐free, uniform composition of thin films. While observing the overall HER activity in terms of their low onset overpotential to encompass a standard current density 10 mA cm^−2^ benchmark in the field of electrocatalysis, ALM‐PtRu thin film is the second best‐performing catalyst concerning the best ALD‐Pt electrode (Figure 7a).

The ALM‐PtRu alloy catalyst only requires ≈30 mV overpotential to reach the 10 mA cm^−2^ benchmark current density, whereas the ALD‐Pt needs 20 mV than that of 58 mV for ALD‐Ru. The strategic control of Pt amounts in ALM‐PtRu alloy could also be noticed and verified from the difference in the obtained overpotentials. Plus, to produce an industrially recommended current density of 100 mA cm^−2^, the ALM‐PtRu alloy only needs 270 mV. For the case of OER, LSV curves were recorded in Figure 7c, and the best‐performing electrocatalyst is ALM‐PtRu film compared to their ALD‐Pt and ALD‐Ru individual stochiometric components. Approximately 50 mV overpotential is required by ALM‐PtRu alloy to reach 10 mA cm^−2^ benchmark current density, which is very low compared to the 440 mV for ALD‐Pt and 110 mV for ALD‐Ru.

A long‐term durability check was performed for ALM‐PtRu alloy for both HER and OER activities, as shown in Figure 7b,d. There is only a negligible overpotential change of ≈20 mV even after 3000 continuous cycles at a fast scan rate. Initial potential and current density maintenance, even after the long electrolysis process, demonstrate the robustness of the developed ALM‐PtRu alloy for further catalysis applications. The demonstrated superior electrocatalytic activities for novel ALM‐PtRu alloy underscore the importance of achieving a homogeneous alloy of PtRu with consistent composition at the nanoscale.

## 3 Conclusion

In the context of the current research endeavor, we successfully introduced homogeneous PtRu alloy thin films utilizing the novel ALM technique, which serves as a viable alternative to the conventional supercycle ALD methodology often employed for achieving multicomponent alloy films. By integrating methodology one, which encompasses the modification of the precursor pulsing duration, with methodology two, which pertains to the control of the precursor temperature, we proficiently adjusted the adsorption properties of the precursor material to achieve significant changes in the resulting alloy composition. Comprehensive analyses conducted through UHR‐STEM EDS, RBS, ToF‐ERD, XRR, and XRD revealed that the first methodology predominantly yielded Pt‐rich thin films (Pt:Ru = 62:38–97:3), whereas the second methodology facilitated the attainment of Ru‐rich compositions (Pt:Ru = 28:72–97:3). This nuanced tuning of the PtRu alloy composition underscores the profound influence that precursor temperature exerts on the GPC and, by extension, on the stoichiometric balance of the ALM‐PtRu alloy itself. Notably, the pivotal advantage of the ALM technique lies in its capability to foster the development of impurity‐free homogeneous PtRu alloy structures, thereby ensuring a consistent and uniform composition of ≈100% step coverage of ALM‐PtRu is found on the high aspect ratio (∼30) 3D trench structures (top width of 125 nm, bottom width of 85 nm). The resultant ALM‐PtRu alloy films exhibited enhanced catalytic performance when compared to their ALD‐Pt, ALD‐Ru elemental counterparts when utilized as catalysts in both the OER and HER, thereby accentuating their considerable potential in applications related to energy conversion and storage. Our findings unequivocally illustrate that the ALM process stands out as an efficacious method for forming alloy thin films, empowering the strategic design of materials explicitly tailored for catalytic functions and advanced semiconductor applications.

## 4 Experimental Section

### Deposition Process and Mechanism of ALM‐PtRu Alloy Thin Films

The deposition of PtRu alloy films was performed using a traveling‐wave ALD reactor (Lucida D200 HT‐DC, NCD Technology) using the ALM process. In this study, the Pt metal‐organic precursor dimethyl‐(*N,N*‐dimethyl‐3‐butene‐1‐amine‐*N*) platinum (C_8_H_19_NPt, DDAP) and the Ru metal‐organic precursor tricarbonyl(trimethylenemethane)ruthenium[Ru(TMM)(CO)₃], both provided by Tanaka Precious Metals, Japan, were used. The DDAP precursor, solid at room temperature, provided first during the ALM process, was vaporized in bubblers heated to 55 or 65 °C. High‐purity N_2_ (99.999%) gas at a flow rate of 100 standard cubic centimeters per minute (sccm) was used as a carrier gas to transport the vaporized Pt precursor to the reaction chamber. The Ru(TMM)(CO)_3_ precursor, liquid at room temperature, provided second during the ALM process, was vaporized in a bubbler cooled to 10 °C and was transported to the reaction chamber using carrier gas to high‐purity N_2_ gas at a flow rate of 50 sccm. The reactant gas used was high‐purity O_2_ (50 with 50 sccm N_2_ co‐dosing) as a reducing gas (co‐reactant) to deposit the PtRu alloy films. The pulsing time of the DDAP precursor was varied, while the ones of Ru(TMM)(CO)_3_ and of O_2_ reactant were fixed at 10 s. After each precursor and reactant pulse, 200 sccm of N_2_ gas was used as a purge gas for 10 s to remove by‐products and excess chemicals from the reaction chamber. The precursor and reactant delivery lines were maintained at 100 °C to prevent unintended condensation of the precursors during transfer. The process was conducted at 225 °C, with PtRu alloy films deposited on thermally grown SiO_2_/Si substrate.

### Advanced Physiochemical Analysis of ALM‐PtRu Alloy Thin Films

Various analytic methods were used to identify the growth characteristics and physical properties of the ALM‐grown PtRu films on the SiO_2_/Si substrates. The phases and crystallinity of PtRu alloy thin films were investigated using grazing incidence angle x‐ray diffraction (GIAXRD; D8 DISCOVERY, Bruker, U.S., and Smart Lab, RIGAKU, Japan) analysis with Cu Kα radiation with a wavelength (λ) of 1.5406 Å, and a grazing‐incident angle of 0.3°. X‐ray reflectivity (XRR) measured the films' thickness and density with the same equipment. Since the densities of Pt and Ru were different, 21.45 and 12.37 g cm^−^^3^, respectively, the overall density of the alloy depends on the relative ratio of the two metals. Therefore, the density measured by XRR can indirectly be used to estimate the relative proportions of Pt and Ru in the alloy. To confirm the alloy composition more precisely, the composition ratio of Pt and Ru was analyzed using Rutherford backscattering spectrometry (RBS; National Electrostatic Corp. (NEC), 6SBH accelerator with 2 MeV He^2+^ α particle source, Korea Institute of Science and Technology). Impurity concentrations in alloy films such as C and O were further examined using time‐of‐flight elastic recoil detection Analysis (ToF‐ERD, NEC‐6SBH accelerator with 7.5 MeV Cl^+^ source, Korea Institute of Science and Technology). The microstructure of the alloy film was analyzed through plan‐view and cross‐sectional view transmission electron microscopy (ultra‐high‐resolution transmission electron microscopy (UHR‐TEM; FEI Themis Z, Thermo‐Fisher Scientific, U.S.). Elemental quantification and distribution were assessed using Scanning TEM (STEM)‐energy dispersive spectroscopy (EDS) with the same TEM equipment. The alloy film's physical and compositional step coverage was evaluated using XTEM and STEM‐EDS analysis by depositing the films on a 3D trench structure with an upper width of 125 nm, a lower width of 85 nm, and an aspect ratio of ≈30.

### ALM‐PtRu Alloy Thin Films's Electrocatalyst Fabrication and Investigation

The possible use of cost‐effective ALM‐PtRu precious metal alloy thin films in both electrocatalytic hydrogen and oxygen production processes was also investigated to ensure the efficient use of any precious metals such as Pt and Ru. Individual ALD‐Pt and ALD‐Ru electrode catalysts were also made in order to have a clear electrochemical comparison concerning the novel ALM‐PtRu atomically dispersed homogeneous alloy film. BioLogic SP‐150e potentiostat/galvanostat electrochemical workstation was used to screen the electrochemical performances of ALD‐Pt, Ru, and ALM‐PtRu catalysts. An electrolyte of 0.5 M H_2_SO_4_ was used, and a constant electrolyte was made for the convenience of all electrocatalyst samples' performance comparison and easy potential (V) conversion versus reversible hydrogen electrodes (RHE). Pt‐counter electrode and Ag/AgCl reference electrode systems were employed for testing the electrocatalyst's durabilities and efficiencies with the measurements, such as linear sweep voltammograms (LSVs) and cyclic voltammograms (CVs).

## Conflict of Interest

The authors declare no conflict of interest.

## Supporting information



Supporting Information

## Data Availability

The data that support the findings of this study are available from the corresponding author upon reasonable request.
