# Supplementary material for: Advanced Atomic Layer Modulation Based Highly Homogeneous PtRu Precious Metals Alloy Thin Films
Source: Adv Sci (Weinh). 2025 May 28;12(29):e03561. doi: 10.1002/advs.202503561 (PMC12362800; doi:10.1002/advs.202503561)
Supplement: Supplementary file 1 — Supporting Information [file ADVS-12-e03561-s001.docx]

Supporting Information

Advanced Atomic Layer Modulation Based Highly Homogeneous PtRu Precious Metals Alloy Thin Films

Yeseul Son, Sang Bok Kim, Debananda Mohapatra, Taehoon Cheon, and Soo-Hyun Kim*

Y. Son, S.B. Kim, D. Mohapatra, S.-H. Kim

Graduate School of Semiconductor Materials and Devices Engineering, Ulsan National Institute of Science and Technology (UNIST), Ulju-gun, Ulsan 44919, Republic of Korea

E-mail: soohyunsq@unist.ac.kr

T. Cheon

Center for Core Research Facilities, Daegu Gyeongbuk Institute of Science and Technology (DGIST), Dalseong-gun, Daegu 42988, Republic of Korea

S.-H. Kim

Department of Materials Science and Engineering, Ulsan National Institute of Science and Technology (UNIST), Ulju-gun, Ulsan 44919, Republic of Korea

**Keywords**: Atomic layer modulation, Atomic layer deposition, Precious metals, PtRu atomic alloy, Homogeneous composition.


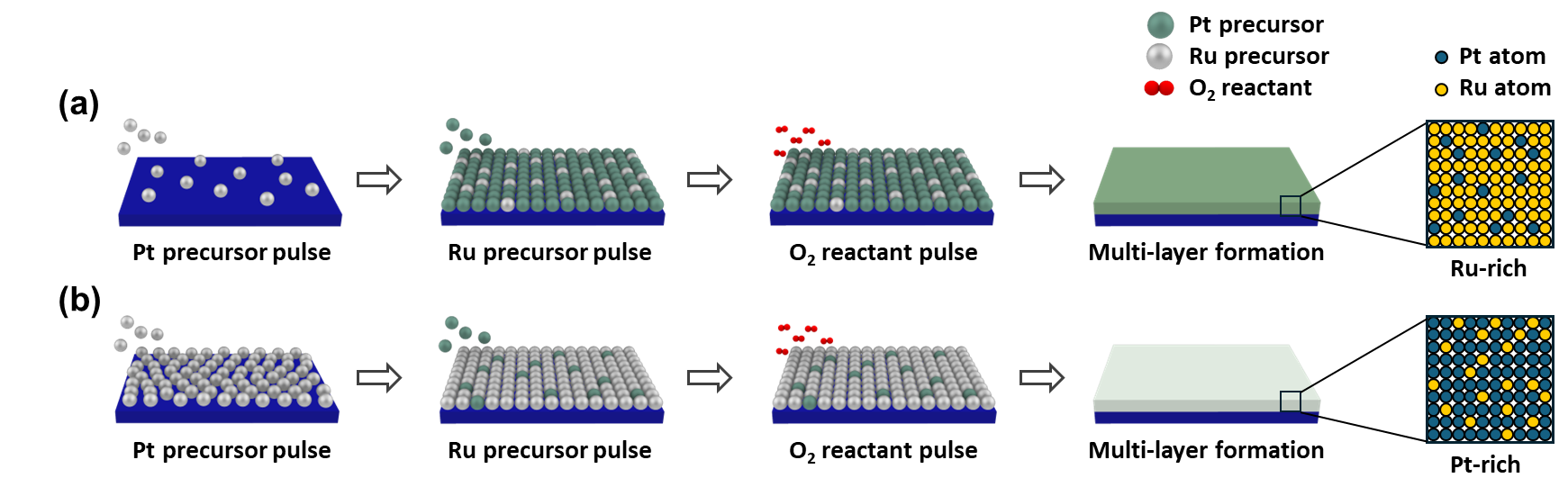


Figure S1. Schematic of ALM process. (a) Pt precursor chemisorption in lower amounts compared to Ru precursor on the surface, resulting in an element Ru-rich alloyed film, (b) Pt precursor chemisorption in higher amounts than Ru precursor, leading to an element Pt-rich alloyed films.


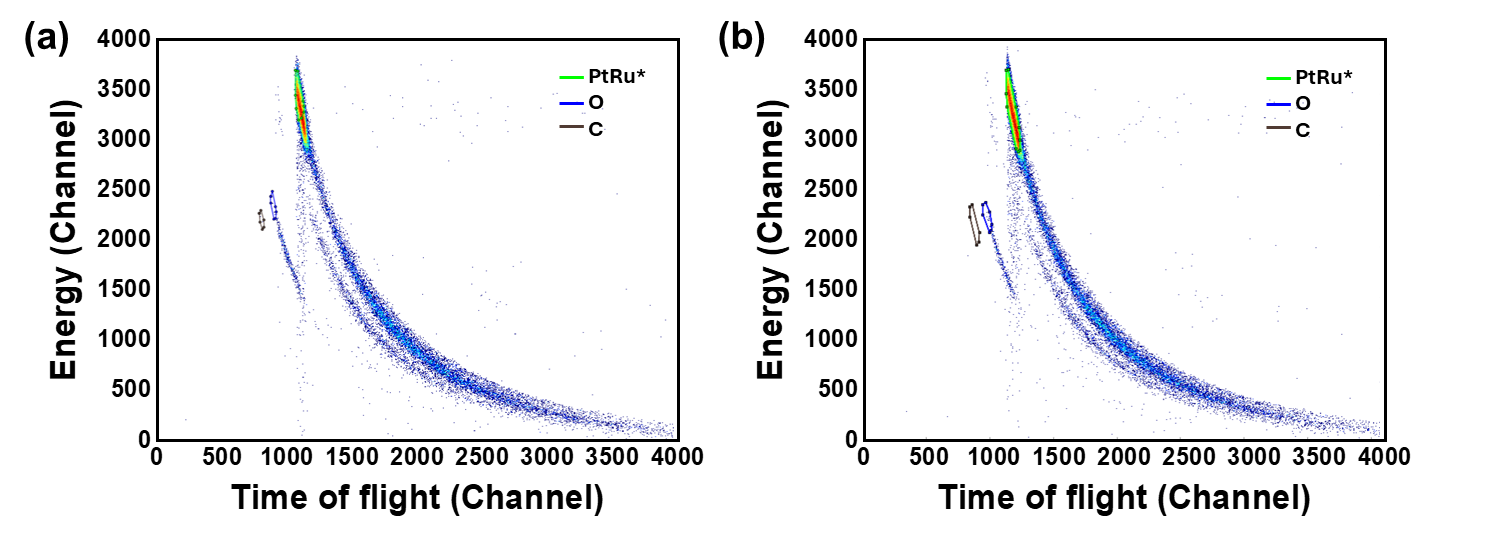


Figure S2. ToF-ERD analysis results of (a) Pt 3-second pulsed and (b) Pt 10-second pulsed PtRu films.

**
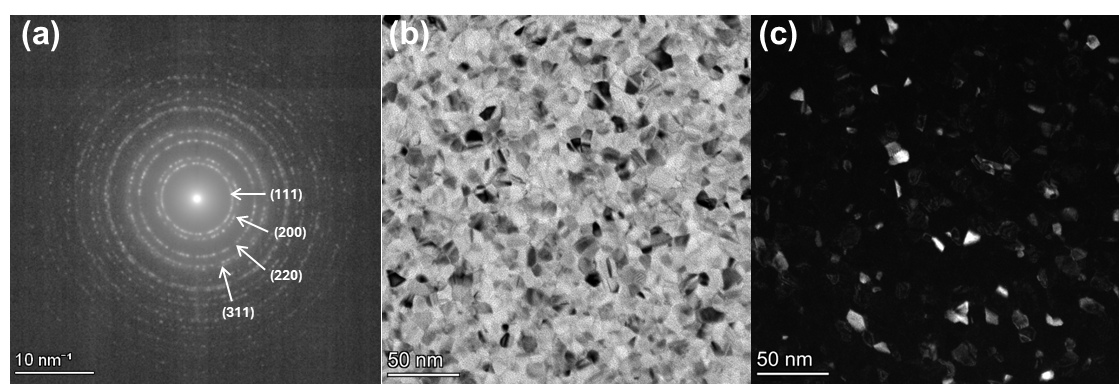
**

Figure S3. Plan view of ALM-PtRu films showing (a) SADP image, (b) bright-field TEM (BF-TEM) image, (c) dark-field TEM (DF-TEM) image of the ALM-PtRu film deposited on a SiO_2_/Si substrate.


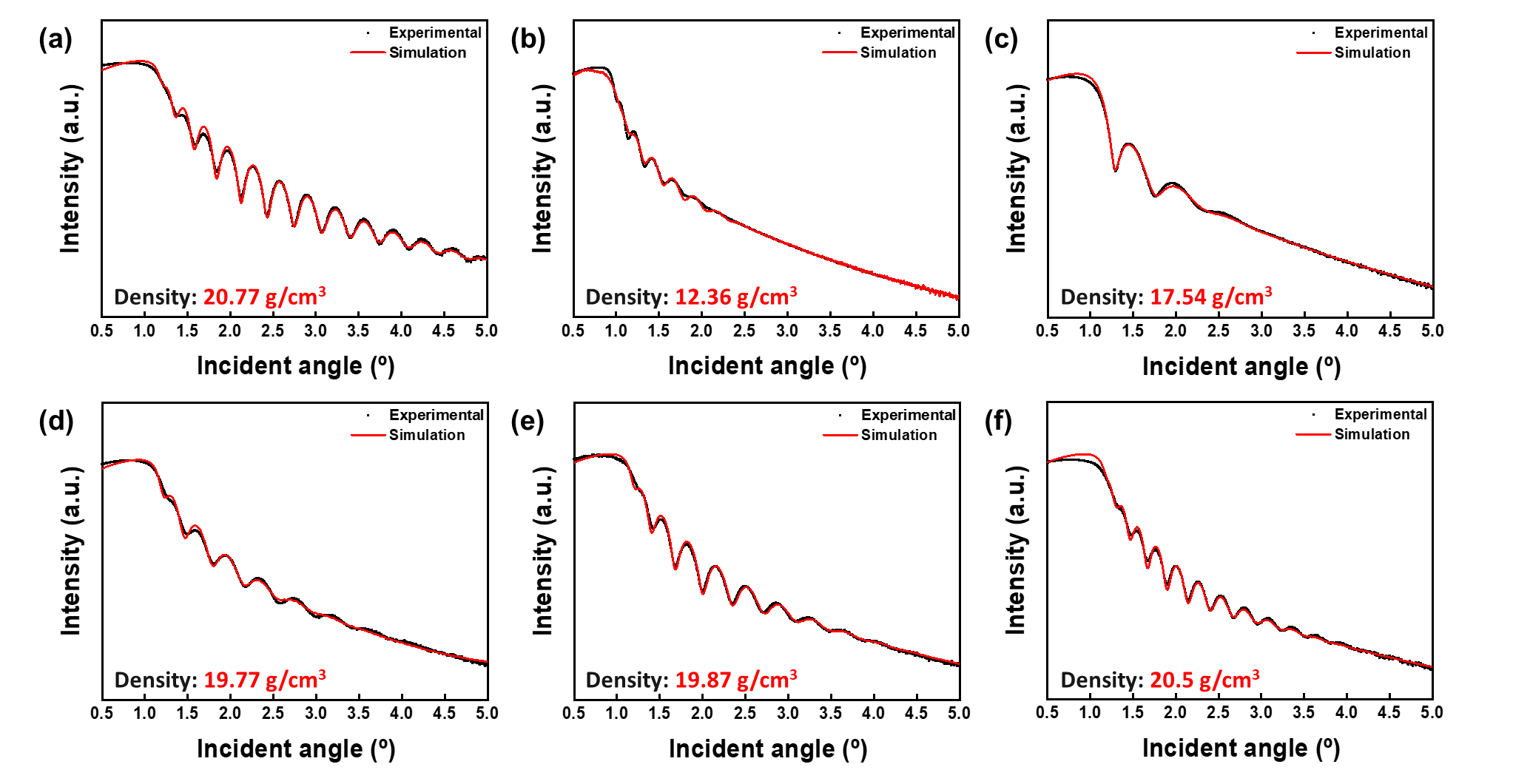


Figure S4. XRR data (experimental and simulated) of ALM-PtRu thin films indicating its approximate density; (a) pure Pt (65 °C), (b) pure Ru, (c-f) PtRu films with Pt 1-, 3-, 5-, 10-second pulsed.


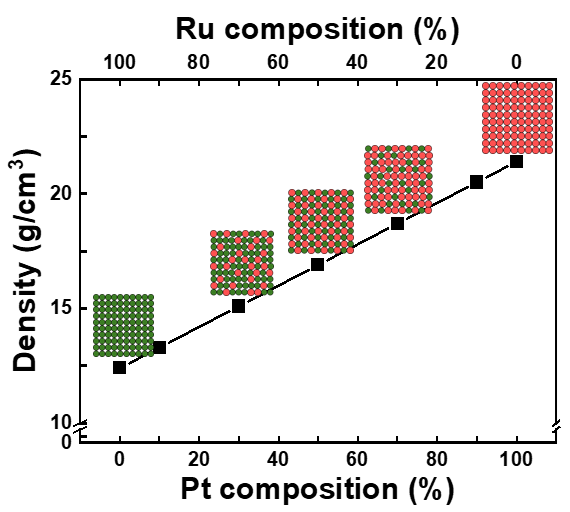


Figure S5. The density of PtRu films as a function of the Pt and Ru composition ratio illustrates the relationship between alloy density and composition.


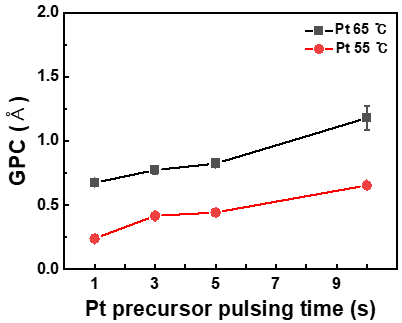


Figure S6. GPC of PtRu films grown on a SiO_2_/Si substrate as a function of Pt precursor pulsing time measured at two different Pt precursor temperatures.


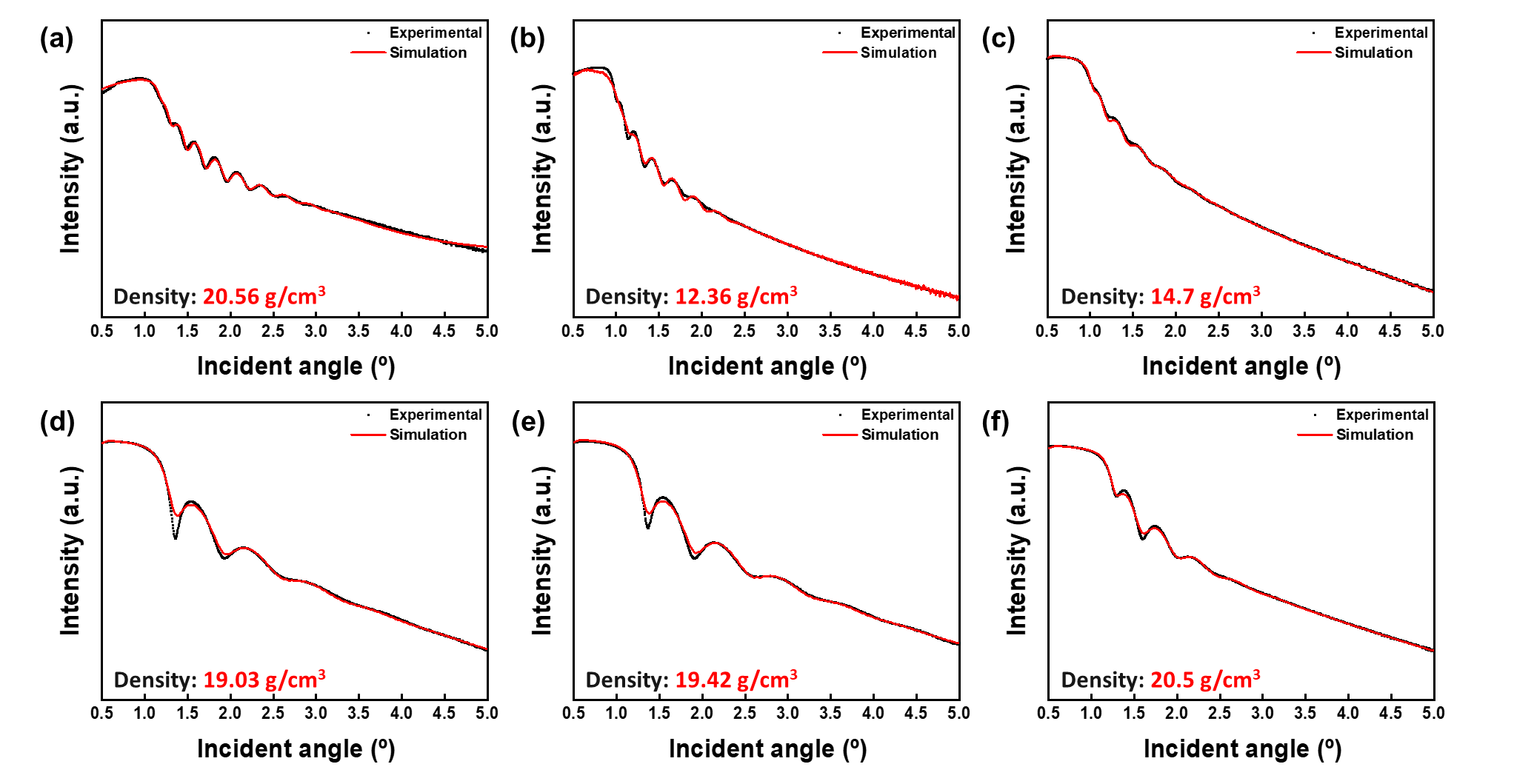


Figure S7. XRR data (experimental and simulated) of ALM-PtRu thin films indicating its approximate density; (a) pure Pt (55 °C), (b) pure Ru, (c-f) PtRu films with Pt 1-, 3-, 5-, 10-second pulsed.


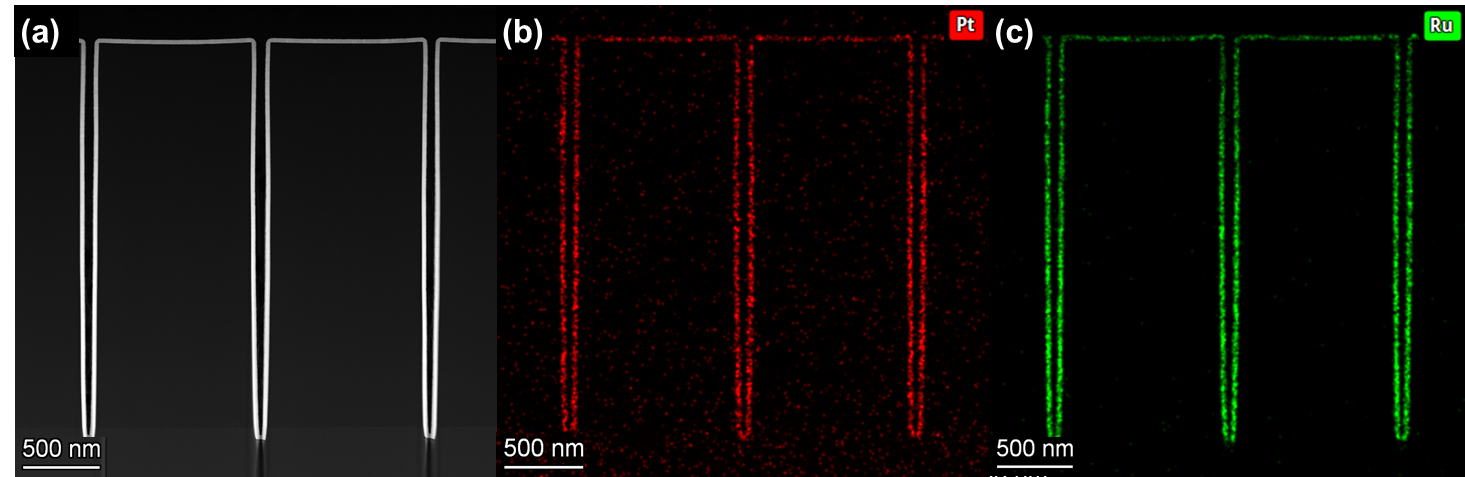


Figure S8. (a) HAADF-STEM image, (b) EDS mapping of Pt, and (c) EDS mapping of Ru of the ALM-PtRu film deposited on a 3D trench wafer.


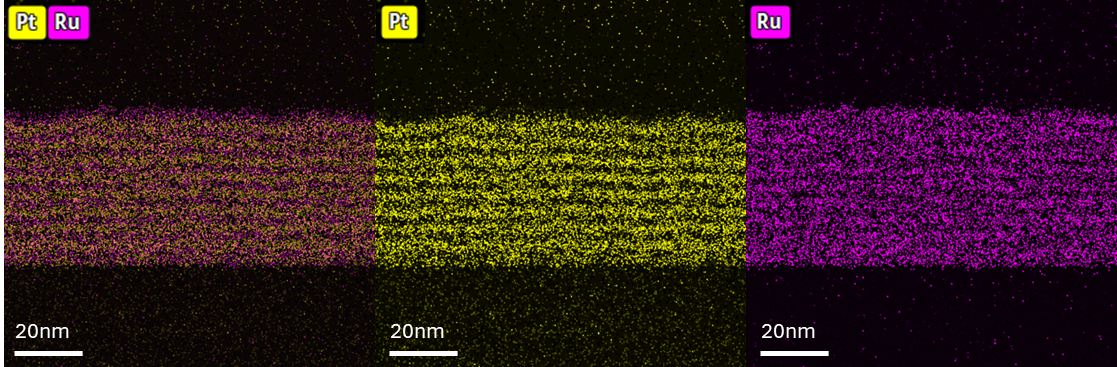


Figure S9. XTEM images of PtRu alloy films prepared by an ALD supercycle method.
